# Case Report: Early rehabilitation initiated in the intensive care unit for myasthenic crisis

**DOI:** 10.3389/fresc.2026.1791183

**Published:** 2026-06-10

**Authors:** Akiyuki Okamoto, Yusuke Sasaki, Yuto Mizutani, Yoshiaki Nakayama, Shoko Yorozu, Kohei Hori, Ken Kouda

**Affiliations:** 1 Department of Rehabilitation Medicine, Wakayama Medical University, Wakayama, Japan; 2 Department of Rehabilitation Medicine, Gifu Municipal Hospital, Gifu, Japan; 3 Division of Rehabilitation, Wakayama Medical University Hospital, Wakayama, Japan; 4 Department of Neurology, Wakayama Medical University, Wakayama, Japan

**Keywords:** case report, early mobilization, mechanical ventilation, myasthenia gravis, overwork weakness

## Abstract

Myasthenia gravis is an autoimmune disorder of the neuromuscular junction characterized by fatigable muscle weakness. Myasthenic crisis is a life-threatening condition characterized by acute respiratory failure resulting from severe weakness of the respiratory muscles. Currently, no established guidelines exist for physiological assessment at the time of mobilization, or for the content and intensity of exercise therapy, in patients with severe myasthenia gravis presenting with myasthenic crisis. We report a case of newly diagnosed myasthenia gravis with myasthenic crisis wherein exercise therapy was initiated in the intensive care unit, with careful monitoring of vital signs and muscle fatigability, ultimately leading to discharge. A 67-year-old woman presented with a dropped head, gait disturbance, and respiratory failure, and was diagnosed with myasthenia gravis at our hospital. On the same day, the patient developed hypoxemia, underwent endotracheal intubation, and was admitted to the intensive care unit for mechanical ventilation. Exercise therapy was initiated on hospital day 3, and the exercise workload progressively increased while vital signs and fatigability were monitored. Standing training began on day 12; and ambulation training while receiving mechanical ventilation was introduced on day 17 without adverse events. The patient was successfully weaned off mechanical ventilation on day 24, and exercise therapy was continued thereafter. Physical function improved after steroid pulse therapy and a thymectomy; she achieved independent ambulation with a cane on day 64 and was discharged on day 78. Early exercise therapy guided by monitoring and muscle fatigue assessments may facilitate functional recovery without clinical deterioration. However, larger studies are required to evaluate its safety and efficacy.

## 1 Introduction

Myasthenia gravis (MG) is an autoimmune disease that affects the neuromuscular junction. It is characterized by fatigable muscle weakness that worsens with repetitive or sustained activity. Myasthenic crisis (MC) occurs in approximately 20%–30% of patients with MG and is typically observed within the first year after symptom onset; however, it may also occur earlier after onset in some patients (1). MC is a life-threatening condition caused primarily by severe respiratory muscle weakness often associated with bulbar dysfunction and airway obstruction, which leads to acute respiratory failure, necessitating mechanical ventilation (invasive or non-invasive) and multidisciplinary management in the intensive care unit (ICU) (2, 3).

Rehabilitation plays an important role in the management of MG. Although standardized guidelines for exercise therapy in patients with MG have not been established, in clinically stable patients with MG, at least 150 min/week of moderate-intensity aerobic and resistance training are recommended, with both safety and efficacy having been reported (4, 5). In contrast, in patients with severe MG complicated by MC, neither clinical assessment criteria at the time of mobilization nor the optimal content and intensity of exercise therapy have been clearly defined. This gap is attributable to the life-threatening nature of MC (e.g., acute respiratory failure) and concerns that exercise may exacerbate MG symptoms, given that fatigability is a core clinical feature of the disease.

Acute exacerbations of MG have been reported to persistently worsen the disease course and contribute to a decreased quality of life (QOL), even after appropriate treatment (6). Indeed, patients who have experienced MC reportedly have lower QOL (7). Accordingly, we hypothesized that, even in patients with MC, exercise therapy implemented under appropriate monitoring may improve physical function without worsening clinical status.

In this report, we describe the clinical course of a patient with newly diagnosed MG who presented with MC; and in whom exercise therapy was actively initiated early during her ICU stay while her vital signs and fatigability were carefully monitored, ultimately resulting in a successful discharge.

## 2 Case presentation

A 67-year-old woman (height, 154 cm; weight, 40.4 kg; body mass index, 16.9 kg/m^2^) was independent regarding activities of daily living (ADL) prior to symptom onset. Three months before transfer (X−3 months), she developed a dropped head and dysphagia. Two months before transfer (X−2 months), independent ambulation became difficult, and she visited a local clinic, where she was diagnosed with dropped head syndrome. Seven days before transfer (Y−7 days), she developed difficulty moving and a decreased level of consciousness at home and was admitted to the referring hospital with a diagnosis of dehydration. Three days before transfer (Y−3 days), hypercapnia was noted, and noninvasive positive pressure ventilation was initiated. On day Y, the patient was transferred to our hospital with fatigable ptosis, proximal muscle weakness, and bulbar symptoms. The ice pack test was positive, and the Harvey–Masland test showed a waning response. Chest CT revealed a nodular lesion in the anterior mediastinum. Based on these clinical and paraclinical findings, the patient was diagnosed with MG. On the same day, impaired oxygenation was observed, and she underwent orotracheal intubation, after which mechanical ventilation was initiated (controlled mechanical ventilation [CMV] mode; positive end-expiratory pressure [PEEP], 5 cmH₂O; fraction of inspired oxygen [FiO₂], 0.5). She was admitted to the ICU on day 2 of hospitalization. That night, FiO₂ was gradually reduced to 0.21, and on day 3, after spontaneous breathing stabilized, the ventilatory mode was changed to pressure support ventilation (PSV).

### 2.1 Exercise therapy protocol and monitoring

**Figure 1:**
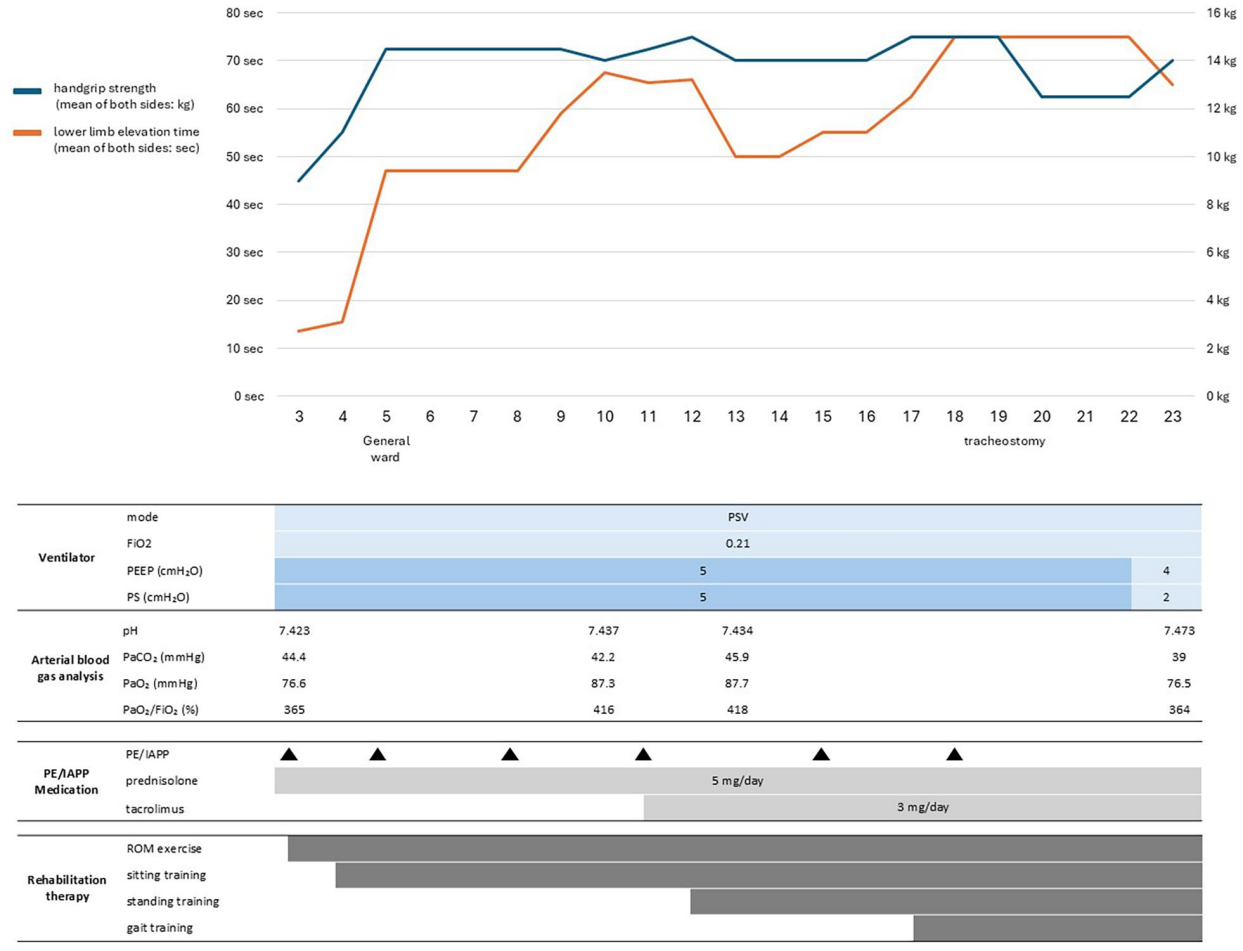
Changes in handgrip strength, lower limb elevation time, ventilator settings, arterial blood gas analysis, and clinical course. FiO₂, fraction of inspiratory oxygen; PEEP, positive end-expiratory pressure; PS, pressure support; PSV, pressure support ventilation; pH, potential of hydrogen; PaO₂, arterial partial pressure of oxygen; PaCO₂, arterial partial pressure of carbon dioxide; RRT, renal replacement therapy; PE, plasma exchange; IAPP, immunoadsorption plasma perfusion; ROM, range of motion.

Figure 1 summarizes the clinical course from initiation of rehabilitation to ventilator weaning. Plasma exchange was initiated on day 2. After confirmation of positivity for anti–acetylcholine receptor (AChR) antibodies, the apheresis strategy was switched to immunoadsorption for a total of seven sessions. Oral corticosteroid therapy was initiated on day 2, and tacrolimus was administered on day 11. The patient was transferred to the general ward on day 5, while mechanical ventilation was continued. Exercise therapy was initiated on day 3. To capture diurnal variations and detect overwork weakness, the intervention time was kept constant each day. Each session lasted approximately 20 min. Before each session, grip strength and duration of leg elevation—components of the Quantitative Myasthenia Gravis (QMG) score—were assessed. On the first day of intervention, therapy was limited to clinical assessment and range-of-motion exercises, and assisted sitting training was introduced the following day. After ICU discharge, mechanical ventilation remained necessary; under PSV with a PEEP of 5 cmH₂O and an FiO₂ of 0.21, the PaO₂/FiO₂ ratio remained stable in the 300–400 range. During sitting training and following exercise therapy, the patient did not exhibit marked tachypnea or reduced tidal volume. Grip strength and leg elevation duration showed an overall trend toward improvement without exacerbation.

**Figure 2:**
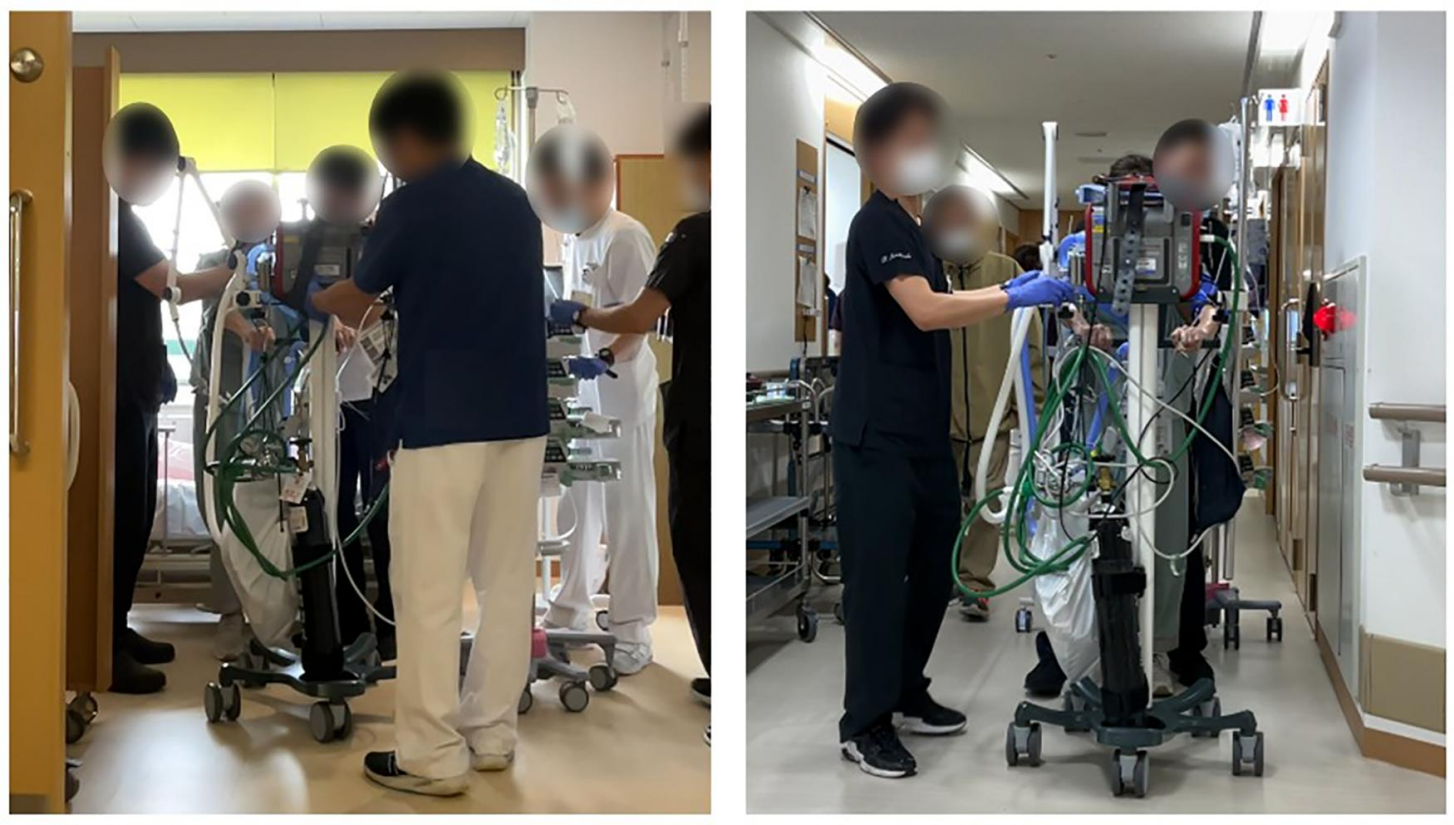
Gait training performed under mechanical ventilation.

Standing training was initiated on day 12 without deterioration in respiratory status. Although the leg-elevation duration transiently decreased on day 13, it subsequently improved. Gait training with the assistance of mechanical ventilation was initiated on day 17 (Figure 2). The patient performed a 10-m walk with a heart rate of 110 beats/min and a maintained tidal volume of ≥ 500 mL, indicating stable and safe performance. Thereafter, the walking distance progressively increased without evidence of clinical worsening, and she was able to walk 70 m continuously by day 23.

**Figure 3:**
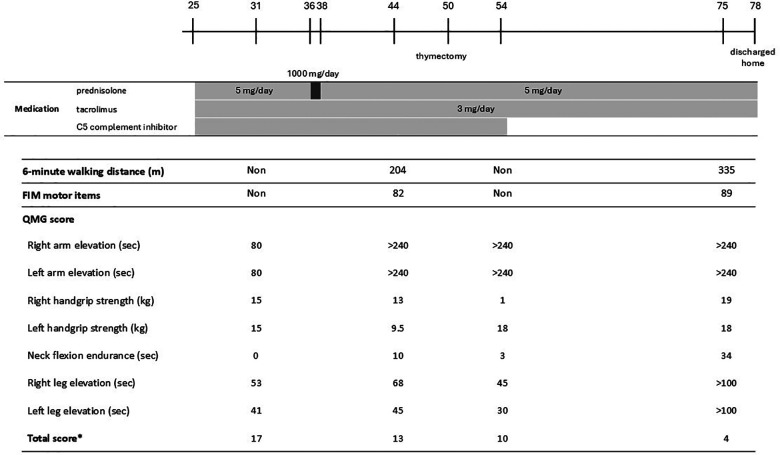
Changes in treatments, physical function, and activities of daily living after weaning from mechanical ventilation. FIM, functional independence measure; QMG, quantitative myasthenia gravis score. *The QMG score was calculated as the total score, excluding %FVC.

Mechanical ventilation was discontinued on day 24. Figure 3 summarizes post-weaning treatments and changes in physical function following ventilator discontinuation. Pharmacotherapy included C5 complement inhibitors and steroid pulse therapy. As a non-pharmacological intervention, thymectomy was performed on day 50. The postoperative course was favorable, and the C5 complement inhibitor was discontinued on day 54. As the patient's respiratory status had stabilized on room air by day 30, exercise therapy was initiated in the rehabilitation gymnasium. The exercise program included standing and gait training, resistance training, endurance training using a lower-limb ergometer, stair-climbing training, and ADL training. Exercise therapy was provided for approximately 60–90 min per day and was adjusted according to perceived fatigue. Resistance training began with body-weight exercises, followed by gradual progression using ankle weights or dumbbells, manual resistance, and a leg press machine. Exercise intensity was titrated using perceived exertion, targeting a Borg Scale rating of ≤ 15; if the Borg Scale reached ≥ 16, the workload was reduced. The target heart rate during standing/gait training and ergometer exercise was calculated using the Karvonen method at an exercise intensity of 70%, and was set at ≤ 140 bpm. After steroid pulse therapy, when cane-assisted ambulation had stabilized, and following extended thymectomy, some components of the QMG score transiently worsened; however, overall physical function improved. The patient was able to ambulate using a walker in the hospital on day 44. By day 57 (postoperative day 7), she was able to ambulate using a cane under supervision. Cane-assisted ambulation became independent on day 64, and the patient was discharged on Day 78. At discharge, the motor subscore of the Functional Independence Measure had improved to 89. After discharge, she continued outpatient pharmacotherapy in the Department of Neurology; the QMG score, excluding respiratory function, remained stable at 3 without exacerbation.

Written informed consent was obtained from the patient for the publication of this case report.

## 3 Discussion

In the present case of MG complicated by MC, exercise therapy was implemented early with careful assessment of clinical status; and physical function was safely restored.

MG causes fatigable muscle weakness resulting from autoantibody-mediated impairment of neuromuscular transmission. Autoantibodies such as anti-AchR and anti-muscle-specific kinase antibodies reduce AchR function at the neuromuscular junction and disrupt the postsynaptic endplate architecture through several mechanisms (8). In contrast, although type II muscle fiber atrophy due to steroid-induced changes has been reported in MG, pathological findings indicative of primary MG-specific structural myofiber abnormality have not been reported (9). Collectively these pathophysiological considerations suggest that, even when neuromuscular transmission efficiency is reduced, muscle metabolic capacity and plasticity are largely preserved. Appropriately titrated exercise loading may therefore contribute to the maintenance and improvement of muscle function.

Several studies have reported the effects of exercise therapy in clinically stable MG. Westerberg et al. reported that a 12-week supervised program of aerobic exercise and strength training improved the QMG scores and lower-limb strength without clinical deterioration (10, 11). Chang et al. reported that, compared with usual care, a 6-week intervention combining moderate-intensity interval inspiratory muscle training and aerobic exercise reduced dyspnea and improved physical function (12). While these studies support the efficacy of exercise therapy in MG, Birnbaum et al. reported symptom exacerbation due to overwork in a patient with MG who participated in a full marathon (13), highlighting the importance of monitoring for overwork weakness when prescribing exercise therapy in MG. Consequently, exercise therapy has traditionally been avoided in severe MG because of respiratory muscle vulnerability, aspiration risk associated with bulbar dysfunction, and heightened risk of general condition deterioration. Wen et al. suggested that exercise interventions should be limited to patients with mild-to-moderate MG (5). However, muscle-wasting progresses rapidly within the first week in critically ill patients admitted to the ICU, and its consequences can be long-lasting (14, 15). Prolonged mechanical ventilation has also been reported to induce disuse atrophy of both the skeletal muscles and diaphragm (16). These issues may be particularly relevant in patients with severe MG, in whom respiratory muscle vulnerability and muscle fatigability are central clinical concerns. In the general ICU population, early rehabilitation has been associated with improved functional outcomes, increased muscle strength, longer walking distance, and enhanced QOL (17).

However, reports on the rehabilitation of patients with MC remain limited. Otaka et al. reported that high-intensity rehabilitation might not contribute to the recovery of ADL in patients with MC (18). However, their study classified rehabilitation intensity solely on the basis of intervention time and did not evaluate the content of the intervention or the actual exercise intensity, which represents an important limitation. In light of these findings, we considered that even in patients with severe MG presenting with MC, it is important to initiate exercise therapy after appropriately evaluating disease activity and general condition, with careful attention to overwork weakness; therefore, exercise therapy was initiated during the ICU stay.

As methods for physiological assessment and determination of optimal exercise intensity during exercise therapy in severe MG complicated by MC have not been established, we limited the initial intervention to sitting training while carefully monitoring for overwork weakness and deterioration in respiratory status. To assess the patient's general condition, vital signs, ventilator settings, and arterial blood gas parameters were monitored. In addition, among the QMG components reported in the Japanese clinical practice guidelines as having high sensitivity for detecting fatigable muscles (19), grip strength—which can be easily assessed at the bedside—and the duration of leg elevation in the supine position, which can be readily measured, were selected. Using these indicators, we were able to capture day-to-day changes in objective measures, muscle fatigue, and stepwise progress to standing and gait training, without the deterioration of her general condition. In this case, the duration of leg elevations decreased on day 13 when standing training was initiated. However, there was no worsening of other objective measures, and the QMG score remained within the mild grade range. Considering that the following day was a non-rehabilitation day, training was continued with the same workload. Thereafter, the leg-elevation duration increased, and no signs of respiratory muscle fatigue emerged. Nevertheless, further investigation is required to determine the degree of worsening in these measures that should be regarded as clinically meaningful deterioration. Similarly, there is no clear consensus on the criteria for discontinuation of exercise therapy. In the present case, although we implemented exercise therapy based on alarm thresholds for the ventilator and monitoring parameters established by the primary team (respiratory rate ≥ 30 breaths/min; tidal volume, 180 mL; SpO₂ < 90%), the establishment of standardized criteria remains a challenge for future research.

Rahbek et al. reported that moderate-to-high-intensity aerobic exercise, combined with progressive resistance training (PRT), was feasible in patients with generalized MG and that the PRT group showed improvements in maximal muscle strength and functional capacity (20). Consistent with these findings, once the patient's MG became clinically stable and gym-based training became feasible after day 30, exercise intensity was progressively increased to 70% of the heart rate reserve, corresponding to moderate-to-high intensity, and an exercise program combining exercise therapy with resistance training was implemented. Following improvement of MC, the patient continued moderate-to-high-intensity resistance training and endurance training without symptom relapse, and both physical function and ADL improved. These findings further support the feasibility of moderate-to-high-intensity exercise in patients with clinically stable MG, in line with the findings of Rahbek et al. (20). In this case, QMG scores and ADL improved over time, and overwork-related weakness was not observed. However, further studies are warranted to optimize the exercise workload and intensity during periods of symptom exacerbation. Moreover, because multiple therapeutic interventions were administered concurrently in this case, additional investigations are needed to clarify the specific contribution of exercise therapy to functional improvement. Furthermore, because quality of life was not assessed in this case, future studies should include quality of life as an outcome measure in addition to physical function and ADL.

In summary, after assessing the patient's vital signs and muscle fatigue, exercise therapy was implemented without a deterioration in her general condition, contributing to improvements in ADL. Although exercise therapy during the MC phase has traditionally been contraindicated, the present clinical course suggests that it may be safely implemented with appropriate assessment and stepwise adjustment of the exercise load. Accumulation of similar cases is warranted to further evaluate the safety and efficacy of early exercise therapy in patients with severe MG.

## 4 Patient perspective

The patient experienced marked anxiety and fear regarding her medical condition and future daily life when her symptoms rapidly worsened, necessitating mechanical ventilation and admission to the intensive care unit. During the course of exercise therapy, she occasionally reported fatigue and frustration but continued to participate in the intervention without refusal. She provided the following account: “The fact that I could gradually move my body became an encouragement and motivated me to continue the intervention.” At the time of discharge, she expressed gratitude for the improvements in her motor function and activities of daily living.

## Data Availability

The raw data supporting the conclusions of this article will be made available by the authors, without undue reservation.
